# Regeneration of a Coastal Pine (*Pinus thunbergii* Parl.) Forest 11 Years after Thinning, Niigata, Japan

**DOI:** 10.1371/journal.pone.0047593

**Published:** 2012-10-16

**Authors:** Jiaojun Zhu, Yutaka Gonda, Lizhong Yu, Fengqin Li, Qiaoling Yan, Yirong Sun

**Affiliations:** 1 State Key Laboratory of Forest and Soil Ecology, Institute of Applied Ecology, Chinese Academy of Sciences, Shenyang, China; 2 Qingyuan Experimental Station of Forest Ecology, Chinese Academy of Sciences, Shenyang, China; 3 Faculty of Agriculture, Niigata University, Niigata, Japan; DOE Pacific Northwest National Laboratory, United States of America

## Abstract

To examine the effects of thinning intensity on wind vulnerability and regeneration in a coastal pine (*Pinus thunbergii*) forest, thinning with intensities of 20%, 30% and 50% was conducted in December 1997; there was an unthinned treatment as the control (total 8 stands). We re-measured the permanent sites to assess the regeneration characteristics 11 years after thinning. In the 50% thinned stand, seedlings aged from 2 to 10 years exhibited the highest pine seedling density and growth. The age composition ranged from 1–3 years with densities of 9.9 and 5.1 seedlings m^−2^ in 30% and 20% thinned stands; only 1-year-old seedlings with a density of 6.1 seedlings m^−2^ in the unthinned stand. Similar trends were found for the regeneration of broadleaved species such as *Robinia pseudoacacia* and *Prunus serrulata*. We speculate that the canopy openness and moss coverage contributed to the regeneration success in the 50% thinned stand, while the higher litter depth and lack of soil moisture induced the regeneration failure in the unthinned stand. The stands thinned at 20% or 30% were less favourable for pine regeneration than the stands thinned at 50%. Therefore, thinning with less than 30% canopy openness (20% and 30% thinned stands) should be avoided, and thinning at higher than 30% canopy openness (50% thinned stand, approximately 1500 stems ha^−1^ at ages 40–50 years) is suggested for increasing regeneration in the coastal pine forest. The implications of thinning-based silviculture in the coastal pine forest management are also discussed. The ongoing development of the broadleaved seedlings calls for further observations.

## Introduction

The coastal forest, as a protective system along the shoreline (sandy soil), represents an important aspect of the natural environment. Generally, the coastal forest can provide some protection, such as reducing damaging winds, obstructing the movement of blown sand and filtering the atmospheric constituents for the local people [1], [2]. Therefore, it is necessary to apply management regimes to establish and sustain the coastal forest to preserve the continuity of the protective or shelter functions.

Japanese black pine (*Pinus thunbergii* Parl.), an evergreen species, is one of the most important tree species in coastal forests of the Japanese islands [3] and in other similar regions such as the shorelines in Shandong Province, China [4], [5], because the tree species can resist pollution, salt and wind effectively [6], [7]. To sustain the shelter functions of coastal pine forests, natural regeneration in accordance with modern sustainable management is considered an effective strategy for the forest ecosystems [8]–[11]. Undoubtedly, thinning is the most important silvicultural measure to promote natural regeneration and maintain the continuity of shelter benefits.

Natural regeneration of *P. thunbergii* requires large enough canopy openness (e.g., 30%) because *P. thunbergii* is a light-demanding tree species [7], [12]. However, it is a dilemma to thin a coastal pine forest because the *P. thunbergii* forest near the sea is vulnerable to wind risk when the stand is thinned for its regeneration [12]–[15]. Forest managers are therefore seeking methods to regenerate the coastal pine forest without interrupting its shelter functions [4], [6], [7], [14]. The large-scale disturbances (e.g., intensely thinning), particularly carried out with inappropriate period and intensity, would either start or accelerate the wind damage and further affect the shelter functions of the coastal pine forest. It is necessary to balance thinning and wind damage in the coastal pine forest to preserve its shelter functions continuously. Therefore, thinning management that can not only promote natural regeneration but can also avoid wind risk is desirable.

To determine the desirable thinning intensity, we conducted a thinning management experiment with random sampling techniques in a coastal pine forest (approximately 40 years old, initial stocking density of approximately 4500 stems ha^−1^) at the shoreline along the Japan Sea in December 1997. After thinning, the fixed sample plots were set up and observations on the wind vulnerability of stands [15] and the effects of gap size induced by thinning on the pine seedling emergence, survival and establishment were conducted within the first four years [13]. One of the findings in the previous publications is that thinning cannot induce wind damage, and the shelter effects of the coastal pine forest, such as windbreak, of obstructing the movement of sand blown and protecting against erosion can be ensured [15]. More important, thinning can produce canopy openness or gaps to provide suitable conditions for the early stage of natural regeneration of *P. thunbergii* [13]. The results that were published in 2003 [13] indicate that although *P. thunbergii* seeds can germinate under the close canopy, the seedlings that emerged from their seeds are unable to survive. The seedlings seem to require a minimum canopy openness of more than 30% to survive and a canopy openness of more than 40% for further developing into saplings [13].

Thinning creates large canopy openness or gaps with great changes in the physical environment. These changes provide regenerating opportunities for tree species that could not establish under a closed canopy [16]–[19]. Differentiation of the responses of a tree species to the amount of canopy openness and to the variations of the physical environment has significant implications for the general model of forest dynamics [20]–[22]. The effects of canopy openness induced by thinning are generally ephemeral when the other disturbances are rare in the forests [12], but the thinning-induced canopy openness plays a key role on the development of forest structure and on forest floor conditions, such as light regime, soil moisture and ground covers [23], [24]. Therefore, the importance of thinning-induced canopy openness has been recognised in studies of forest regeneration dynamics and seedling or sapling growth phases [13], [25]–[27].

The objective of this study was to examine whether, at the 11^th^ year after thinning treatment, the survival and growth of *P. thunbergii* seedlings were affected by the canopy openness, ground covers and the age of thinning treatment. In addition, we were interested in confirming whether the current regeneration conditions were consistent with the ones predicted at the 4^th^ year and the 6^th^ year after the thinning treatment.

## Materials and Methods

### Site Description and Thinning Treatments

**Figure 1:**
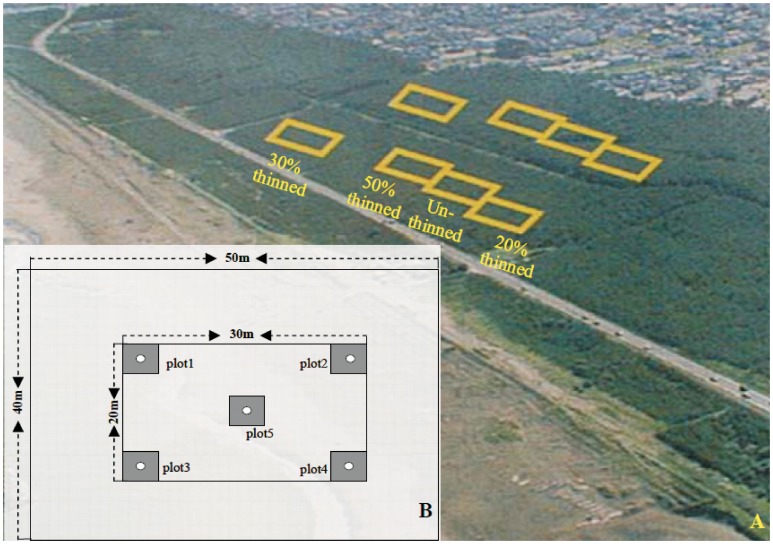
Layout of the permanent experimental site. A: for the general view, B: for the plots. Rectangles: the four thinning treatments with 40 m×50 m for each treatment, 2 repetitions; black square areas: monitoring of seedling regeneration through plots 1–5 (plot area: 2 m×2 m), circles: making canopy openness measurements in the four treatments. The survey of ground covers (litter depth, grass coverage, moss depth and moss coverage) was conducted randomly (avoiding the seedling monitoring plots).

[LOOSEST ]The observations were conducted in the coastal forest at Aoyama coastal area, Niigata prefecture (37°52′41.3′′N, 138°56′16.8′′E), in the middle of the shoreline along the Japan Sea. The non-commercial coastal forest belongs to the local government, Niigata prefecture. Anyone can access the coastal forest and do observations without damaging the trees there. The thinning was authorised by the Department of Forestry of Niigata prefecture when the experiment was started in 1997 [14], [15]. The climate is of an oceanic monsoon type with a windy spring, warm and humid summer, and a snowy winter. The main constraint for the coastal forest is the salty wind. The annual precipitation is 1778 mm; the annual average temperature is 13.2°C ; the minimum temperature is −13.0°C; the first frost is on 24 December, and the last frost is on 30 March [12]. The coastal forest, composed of pure *P. thunbergii*, was planted on the coastal sandy soil with a slope of 4° during the 1960s at spacing of 1.5×1.5 m (the initial stocking density was approximately 4500 stems ha^−1^) and without an understory because of the closed canopy. The width of the coastal pine forest ranged between 100 m and 260 m (**Fig. 1**). The micro-topography in the experimental area was almost the same throughout a wide range. The preserved rate (the ratio of the current stocking density to the initial planting stem density) was averaged at approximately 69% in 2008 because of the effects of self-thinning [13], [28]. There are some broadleaved tree plantations in the landside of the coastal forest. As the natural regeneration in the coastal pine forest is poor [14], [29] and the coastal pine forest is vulnerable to wind risk, a thinning experiment was carried out in December 1997 to test the stand stability against wind damage and the natural regeneration after thinning. The coastal pine forest stands were thinned by basal area with random sampling techniques. The thinning treatments were set up as 20, 30, 50% (thinned) and 0.0% (unthinned) [13]. The effective area of each treatment was 40 m×50 m with one repetition even though the coastal pine forest stands are distributed evenly within a wide range (total 8 stands or plots). The observations were conducted within a 20 m×30 m section in each plot (**Fig. 1**). The general characteristics of the coastal pine forest stands were surveyed before, soon after and 11 years after thinning (**Table 1**).

**Table 1:** Stand characteristics before and after thinning on the basis of plot to plot.

Thinningtreatment	Density (trees ha^−1^)			Basal area (m^2^ ha^−1^)			DBH (cm) (mean ± SD)			Tree height (m) (mean ± SD)		
	BT	AT1	AT2	BT	AT1	AT2	BT	AT1	AT2	BT	AT1	AT2
0%unthinned	3600	3600	3383	23.2	23.2	34.3	8.7±2.2	8.7±2.2	10.8±3.5	6.2±0.9	6.2±0.9	9.5±1.1
20%thinned	3217	2517	2300	23.4	18.8	26.9	9.2±2.7	9.4±2.7	11.5±4.3	7.5±1.6	7.5±1.5	10.6±1.1
30%thinned	3167	2100	1983	21.4	14.5	25.4	9.0±2.2	9.1±2.3	12.3±3.7	5.9±0.8	5.9±0.8	8.6±2.0
50%thinned	3000	1483	1467	26.0	12.9	23.6	10.1±3.1	10.1±3.0	13.5±5.2	7.3±1.5	7.2±1.4	10.1±1.2

Note: BT: before thinning in December 1997; AT1: soon after thinning in February 1998; AT2: 11 years after thinning in October 2008.

SD: standard deviation.

### Measurements

#### Canopy openness

The canopy openness was monitored twice in the growing seasons (August, October) of 2003 and 2008, at the same points as in 2000 when the canopy openness was first monitored (**Fig. 1**). The canopy openness was estimated from the silhouettes of hemispherical photographs using a digital hemispherical camera (Nikon, Coolpix 910, f = 7–21 mm) with a 180° fish-eye converter (Nikon, FC-E8, f = 8–24 mm). The hemispherical images were obtained at a height of 1.0 m above ground at five points for each thinned treatment (Circle in **Fig. 1**) after sunset or before sunrise. The direct light and diffuse light, representing the light conditions in each treatment, were obtained according to the process developed by Steege [30] using Gap Light Analyzer software (GLA Version 2) [31].

#### Soil water content

The soil water contents in three profiles, i.e., 0–10 cm, 20–30 cm and deeper than 50 cm, were measured in each treatment in August 2003 and October 2008 to confirm whether the changing trends of soil moisture were consistent with the results observed before [13], [14]. The soil samples (three repeated) from four soil profiles (9 soil samples for each treatment) were randomly collected from each treatment (avoiding the seedling monitoring plots) using a 5 cm diameter soil corer during the dry period. The surface litter and organic content were removed in the field. The aluminium boxes with the soil samples were wrapped using adhesive tape to avoid evaporation and then dried at 105°C for 24 h in the laboratory to calculate the soil water content.

#### Ground covers

The ground covers, including litter depth (needles and branches), grass cover, and moss cover, were observed in respective five random 2 m×2 m quadrats in 2008. Moss cover did not appear in 2003; therefore, only grass coverage and litter depth were observed in 2003. Grass and moss coverage were estimated by a visual method. The litter and moss depth was measured by a ruler. The data presented in this paper are the mean values of the 5 sample points.

#### Regeneration census

Five quadrats of 2 m×2 m were set up in each treatment to monitor seedling survival and growth (**Fig. 1**). In each quadrat, the number of survival seedlings (note: emergence was considered as 1 year old) was recorded in 2003. The detailed investigation of the survival number, age and growth for seedlings of both *P. thunbergii* and broadleaved tree species was conducted in 2008. At the same time, the stem base diameter and height of seedlings older than 1 year were measured. The age of a *P. thunbergii* survival seedling was identified by counting its whorls because we observed that the pine seedlings do not exhibit polycyclism. The seedling age of the broadleaved tree species was determined by the following steps. First, we measured the seedling height and stem base diameter outside the 20 m×30 m sample plot (**Fig. 1**) and then cut down the seedlings and counted the rings of the stem base diameter. The relationship between the age and the seedling height or stem base diameter outside the 20 m×30 m sample plot was established. Second, the seedling height or stem base diameter within the 20 m×30 m sample plot (**Fig. 1**) was measured. The seedling age of the broadleaved tree species can be determined according to the relationship between the age and the seedling height or stem base diameter. The height of each seedling was determined by measuring the distance from the forest floor (soil surface) to the seedling top.

### Data Analysis

The numbers of survival seedlings, the ground covers and the light condition did not follow the normal distributions. Therefore, the Kruskal-Wallis test (K-W test) with Man-Whitney multiple comparison was used to test the difference of the observations among the four thinned treatments (SPSS software, 16th edition, Chicago, USA). A regression analysis was used to determine the relationships between growing characteristics (including height and stem base diameter) of established seedlings and their ages among the thinned treatments (Microsoft Office Excel 2003).

## Results

### Canopy Openness or Light Condition

Compared with 2000, the canopy openness at 1.0 m above the ground increased in 20% thinned and unthinned treatments but decreased in the most intensely thinned treatment for both 2003 and 2008 (**Table 2**). The canopy openness at 20% thinned and unthinned stands showed a similar increasing trend, i.e., canopy openness increased significantly from 2000 to 2003 (*p*<0.05) but showed no significant variations from 2003 to 2008 (*p*>0.05). The canopy openness of the unthinned stand increased from 8.5% in March 2000 to 18.4% in 2003 and remained stable at 18.7% in 2008. The canopy openness of 30% thinned stands maintained a steady increase with no significant difference during the monitoring (*p*>0.05). However, canopy openness decreased linearly in the 50% thinned stand (from 33.1% in 2000 to 29.1% and 25.2% in 2003 and 2008, respectively). The direct light, diffuse light and total light followed the same trend as the canopy openness in each thinned treatment (**Table 2**).

**Table 2:** Mean values of canopy openness, direct light and diffuse light at a height of 1.0 m in each treatment.

Attributes	Treatment 1(20% thinned)		Treatment 2(30% thinned)		Treatment 3(50% thinned)		Treatment 4(un-thinned)	
	Mean	SD	Mean	SD	Mean	SD	Mean	SD
**March 01 2000** *								
Canopy openness (%)	15.7^aA^	1.3	18.9^aB^	1.5	33.1^aC^	1.9	8.5^aD^	1.1
Direct light (Wm^−2^)	51.4	11.4	133.8	12.1	357.4	14.1	88.9	15.6
Diffuse light (Wm^−2^)	780.5	23.2	940.9	32.0	1058.3	30.9	532.6	19.3
Total light (Wm^−2^)	831.9	31.5	1073.7	30.2	1415.7	33.3	621.5	28.4
Percent of direct light (%)	6.3	1.6	16.4	0.9	43.80	1.8	10.9	1.8
**August 22 2003**								
Canopy openness (%)	20.6^bA^	0.9	19.2^aA^	0.6	29.1^bB^	1.2	18.4^bA^	0.9
Direct light (mol m^−2^ s^−1^)	8.4	1.2	9.0	0.9	10.3	0.8	8.3	0.8
Diffuse light (mol m^−2^ s^−1^)	6.1	0.1	6.3	0.6	6.8	0.2	5.7	0.3
Total light (mol m^−2^ s^−1^)	14.5	1.1	15.3	0.3	17.1	0.6	14.0	0.7
Percent of direct light (%)	41.0	5.8	43.4	4.2	51.2	3.1	40.4	3.8
**October 28 2008**								
Canopy openness (%)	23.0^bA^	0.9	22.5^aA^	1.1	25.2^cA^	1.5	18.7^bB^	0.8
Direct light (mol m^−2^ s^−1^)	8.0	1.4	7.0	0.5	8.7	0.5	6.7	0.6
Diffuse light(mol m^−2^ s^−1^)	6.6	0.3	6.4	0.2	7.0	0.2	5.5	0.2
Total light (mol m^−2^ s^−1^)	14.6	1.3	13.4	0.3	15.7	0.4	12.3	0.8
Percent of direct light (%)	39.1	6.8	34.3	2.3	44.1	1.2	32.9	2.9

* data on March 01 2000 were published in Zhu *et al*. (2003a). 1 mol m^−2^ s^−1^ = 0.0864 Wm^−2^.

SD: standard deviation on the basis of plot to plot.

Data followed by different lowercase letters in columns (comparison between three periods after thinning) and by different capital letters in rows (comparison between four thinning treatments) are significantly different at level *p*<0.05 according to the result of the Kruskal-Wallis test with Man-Whitney multiple comparison.

### Soil Moisture

**Figure 2:**
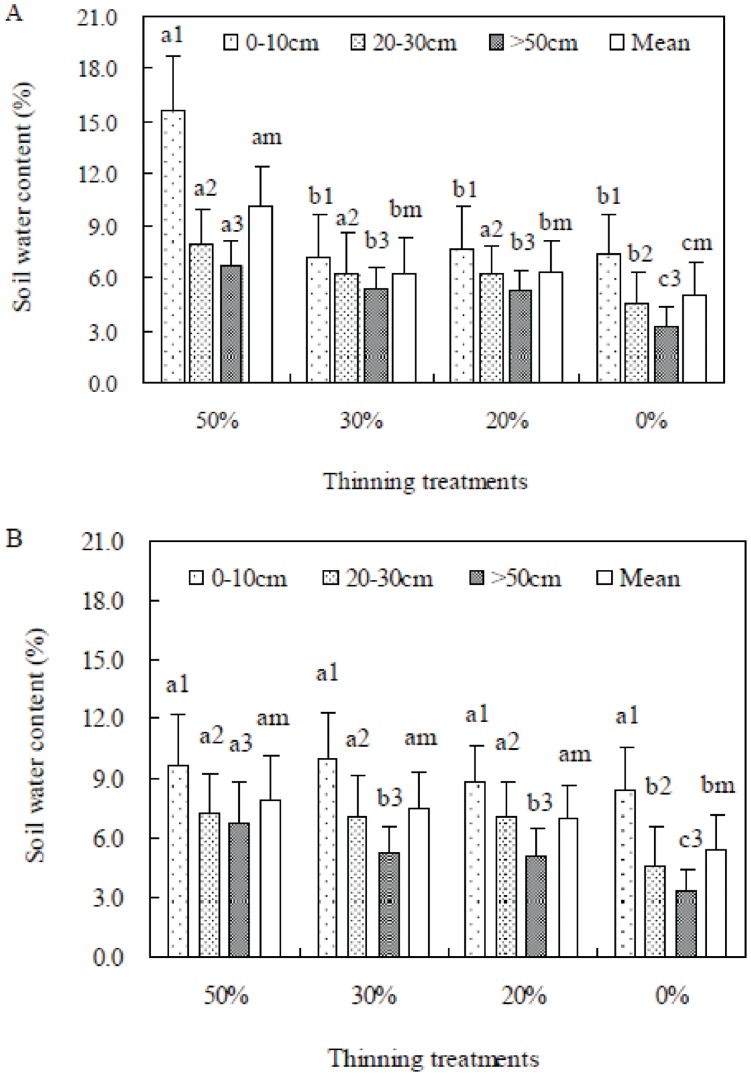
Soil water content at different depths for each treatment in August 2003 (A) and October 2008 (B) on the basis of plot to plot. The same letter followed by a different number indicates the significant difference between thinning treatments at different depths (*p*<0.05).

In August 2003, the soil water content in the 0–10 cm layer of the 50% thinned stand was significantly higher than in other treatment stands (*p*<0.05). For the layers of 20–30 cm, more than 50 cm and the average of soil water content, there were no significant differences among the three thinned stands (*p*>0.05), but significant differences were found between the thinned stands and the unthinned stand (*p*>0.05) (**Fig. 2A**). In October 2008, the water content of layers 20–30 cm, more than 50 cm and the average of soil water content showed a similar trend to the one observed in August 2003. However, there were no significant differences among the four treatments for the soil water content in the 0–10 cm layer (*p*>0.05) (**Fig. 2B**). The changing trends of soil moisture in each treatment were consistent with the observations in 2000 [12].

### Ground Covers

**Figure 3:**
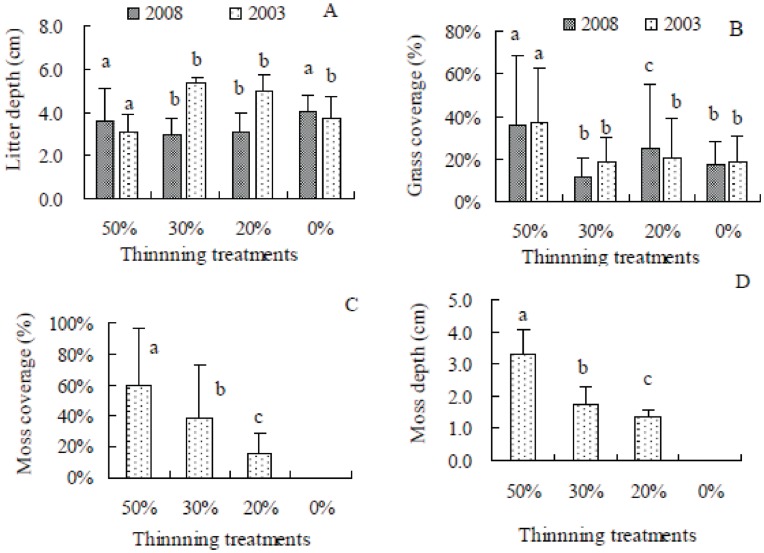
The ground covers for each treatment in 2003 and 2008 on the basis of plot to plot. **A**: litter depth; **B**: grass coverage; **C**: moss coverage in 2008; **D**: moss depth in 2008. The same letters above the histograms of the same year indicate no significant difference between the thinning treatments at level *p*<0.05 according to the result of the Kruskal-Wallis test with Man-Whitney multiple comparison (n = 5).

The litter depth in the 50% thinned stand was significantly lower than in other treatment stands in 2003 (**Fig. 3A**), but the opposite situation occurred for grass coverage (i.e., the grass coverage reached the highest in the 50% thinned stand) (*p*<0.05) (**Fig. 3B**). There were no significant differences of both litter and grass coverage between the other three treatment stands (**Fig. 3A, B**). The litter depth in the 50% thinned stand was significantly higher than in the other two thinned stands but was similar to the unthinned stand in October 2008 (**Fig. 3A**). However, the litter amount in the 50% thinned and unthinned stands did not change between August 2003 and October 2008. The litter depth in the 20% and 30% thinned stands decreased 37.9% and 45.2%, respectively, from 2003 to 2008 (**Fig. 3A**). There was a similar varying trend in grass coverage between 2003 and 2008 (**Fig. 3B**). Besides grass cover and litter cover, moss cover was found in the thinned stands in 2008. Both the moss coverage and moss depth significantly increased with the thinning intensities, and no moss cover appeared in the unthinned stand (**Fig. 3C, D**).

### Regeneration Status

#### Pine regeneration

**Figure 4:**
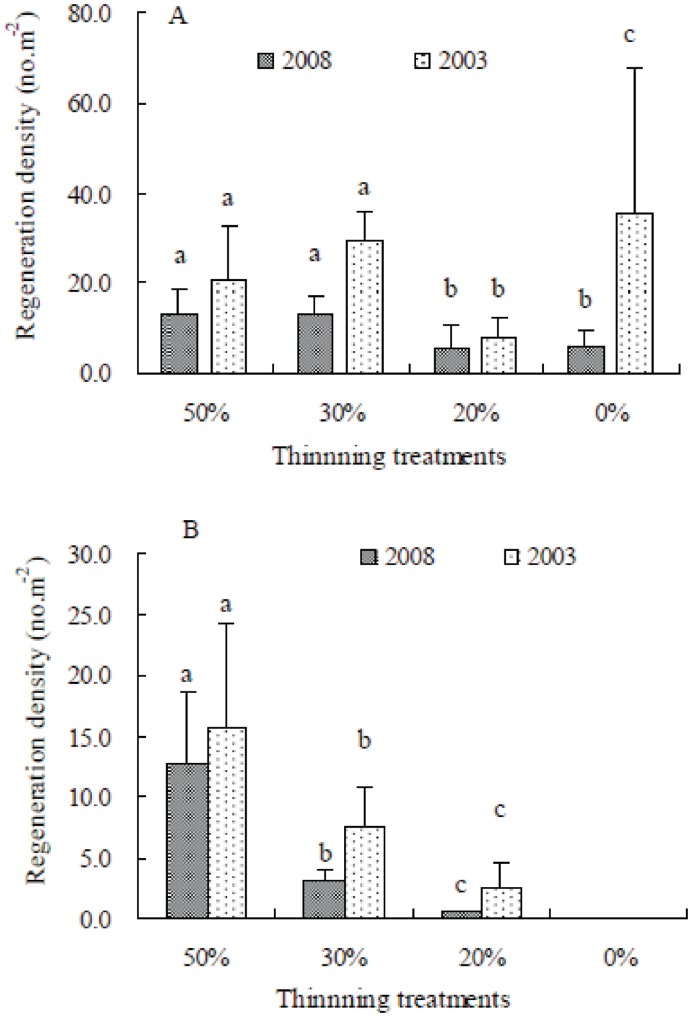
Regeneration density (no. m^−**2**^) for each thinning treatment observed in August 2003 and in October 2008 on the basis of plot to plot. A: the total number of regenerated seedlings, B: the number of regenerated seedlings greater than 1 year old. The same letters above the histograms of the same year indicate no significant difference between the thinning treatments at level *p*<0.05 according to the result of the Kruskal-Wallis test with Man-Whitney multiple comparison (n = 5).

Regenerated pine seedling density ranged from 7.6±4.8 to 35.4±32.1 seedlings m^−2^ in 2003 and from 5.5±5.1 to 12.8±6.0 seedlings m^−2^ in 2008. In 2003, the highest regenerated seedling density occurred in the unthinned stand, and the lowest occurred in the 20% thinned stand. No significant difference in seedling density was found between the 30% and 50% thinned stands (**Fig. 4A**). In 2008, the regeneration densities in the 20% thinned and unthinned stands were significantly lower than those in the 30% and 50% thinned stands (**Fig. 4A**). The age composition of the regenerated seedlings ranged from 2 years to 10 years in the 50% thinned stand and from 1 year to 3 years in the 20% and 30% thinned stands in 2008. All of the regenerated seedlings in the unthinned stand were 1 year old, which was the same as was observed in 2001 [12] and 2003 (**Fig. 4B**). The seedlings more than 5 years of age only occurred in the 50% thinned stand, and the seedling density reached 1.18 seedlings m^−2^. The regenerated seedling density in each treatment in 2003 was higher than in 2008 because of more 1- year-old or 2-year old seedlings in 2003 than in 2008 (**Fig. 4**).

#### Regeneration of broadleaved tree species

The regeneration of broadleaved seedlings was found during the investigation in 2003, but the seedlings were not recorded because of the low number. Four broadleaved tree species, i.e., *Robinia pseudoacacia*, *Rubus* sp., *Prunus serrulata* and *Elaeagnus multiflora*, regenerated in the four treatment stands in the survey of 2008. The regenerated seedling density ranged between 0.18 and 0.36 seedlings m^−2^ at 11 years after thinning (i.e., in 2008) (**Table 3**). Three broadleaved species (*R. pseudoacacia*, *Rubus* sp. and *Prunus serrulata*) appeared in the 50% thinned stand of which more than 80% of the regenerated seedlings were *R. pseudoacacia*. The regenerated seedling density was significantly higher in the 50% thinned stand than in the other treatment stands. In the other three treatment stands, two broadleaved species appeared, and more than 80% of the regenerated seedlings were *Prunus serrulata*. The seedling age of the regenerated broadleaved species ranged from 1 to 10 years in the 50% thinned stand, from 1 to 4 years in the 30% thinned stand, and from 1 to 2 years in the 20% thinned and unthinned stands (**Table 3**).

**Table 3:** Survival and growth of regenerated seedlings for broadleaved species at the 11^th^ year after thinning in four treatments.

Age*	Seedling number	Seedling height (cm)	SD	Stem base diameter (cm)	SD	Density (no. m^−2^)
50% Thinned						
1	3	10.0	1.0	0.17	0.03	
2	3	21.3	9.5	0.23	0.08	
3	3	29.0	10.0	0.27	0.12	
4	2	51.0	25.5	0.50	0.28	
5	1	89.0		0.50		
6	3	91.0	12.7	0.70	0.14	
10	1	190.0		1.80		
Sub-total	16 (3 species, 13 *Robinia pseudoacacia*)					0.36^b^
30% Thinned						
1	5	12.0	0.5	0.11	0.01	
2	4	18.5	0.7	0.20	0.00	
4	1	43.0		0.40		
Sub-total	10 (2 species, 7 *Prunus serrulata*)					0.23^a^
20% Thinned						
1	5	11.0	7.8	0.26	0.15	
2	3	25.0	18.4	0.28	0.18	
Sub-total	8 (2 species, 7 *Prunus serrulata*)					0.18^a^
0% Unthinned						
1	4	11.8	9.4	0.24	0.07	
2	6	8.0	3.6	0.20	0.10	
Sub-total	10 (2 species, 9 *Prunus serrulata*)					0.23^a^

Data not followed by the same letter in the Density column are significantly different at level *p*<0.05 according to the result of the Kruskal-Wallis test with Man-Whitney multiple comparison.

SD: standard deviation on the basis of the same aged seedlings.

Note*: age was determined according to the regression of age- seedling height (H) because the regression of age- H was better than that of age-stem base diameter (D). age  = 0.0493H +0.9916, R^2^ = 0.9577, H ranged between 8 cm and 190 cm. Number of sample: 32 (1-year old: 11, 2-year old: 6, 3-year old: 3, 4-year old: 3, 5-year old: 2, 6-year old: 3, 8-year old: 2, 10-year old: 2).

The relationship between age and stem base diameter was also established: age  = 3.4525 ln(D) +7.1213, R^2^ = 0.9018.

### Growth of the Regenerated Seedling

**Figure 5:**
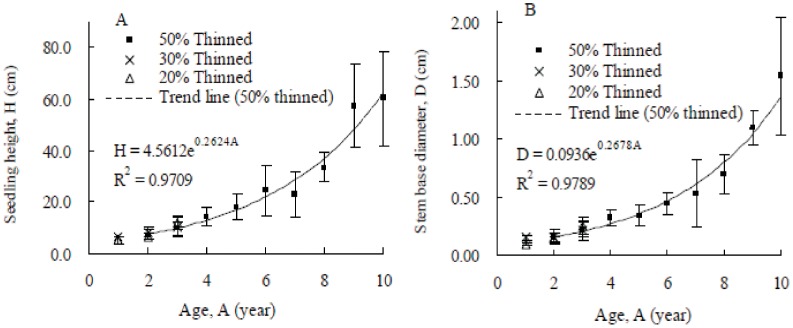
Growth of the established pine seedlings in October 2008 on the basis of plot-to-plot. A: Height growth, B: Stem base diameter growth. The vertical bars indicate the standard deviation.

Seedling establishment had obviously failed in the unthinned stand; therefore, the measurement of seedling growth was only performed in the thinned stands in 2008. Both height and stem base diameter of pine seedlings increased exponentially with age in the thinned stands (**Fig. 5**). There were no significant differences among the growth of 2 and 3-year-old pine seedlings in the thinned stands (*p*>0.05). The growth of older pine seedlings in the 50% thinned stand exhibited an increasing trend, e.g., the mean height and stem base diameters of 8, 9 and 10-year-old seedlings were 33.5±5.9 and 0.70±0.16 cm, 57.5±16.3 and 1.10±0.14 cm, and 60.5±18.3 and 1.55±0.51 cm, respectively (**Table 4**). It should be noted that the mean height of the pine saplings slowed from 9 years (57.5±16.3) to 10 years (60.5±18.3), i.e., the growth rate was only 3.0 cm per year (**Fig. 5**). Similar to the growth trend of pine seedlings, the broadleaved seedlings also showed the highest growth in the 50% thinned stand (**Table 3**).

## Discussion

### Pine Regeneration

Eleven years after the thinning, establishment is successful in the thinned stands, but the regenerated density and the growth of seedlings in the 50% thinned stand were significantly higher than those in the other two thinned stands (**Fig. 4**, **Table 4**). The light regime and the ground covers in the treatment stands may contribute to these results.

**Table 4:** Survival and growth of regenerated *P. thunbergii* seedlings at the 11^th^ year after thinning in four treatments (n = 5).

Age	Sample number of seedlings	Density (no. m^−2^)	Seedling height (cm)	SD	Stem base diameter (cm)	SD
50% Thinned						
2	97	5.70	7.2	1.9	0.15	0.04
3	31	1.82	9.8	2.9	0.22	0.07
4	48	2.82	14.4	3.7	0.32	0.08
5	21	1.23	18.1	5.2	0.34	0.09
6	17	0.41	24.7	9.9	0.44	0.10
7	7	0.18	23.0	8.9	0.53	0.29
8	10	0.24	33.5	5.9	0.70	0.16
9	5	0.12	57.5	16.3	1.10	0.14
10	10	0.24	60.5	18.3	1.55	0.51
30% Thinned						
1	85	9.91	6.4	0.1	0.15	0.02
2	65	3.00	7.9	2.6	0.16	0.07
3	5	0.11	10.3	3.9	0.21	0.09
20% Thinned						
1	68	5.09	5.3	1.2	0.10	0.01
2	17	0.39	6.8	1.3	0.14	0.02
3	2	0.05	12.0	2.7	0.25	0.07
0% Unthinned						
1	80	6.11	5.5	1.0	0.12	0.03

SD: standard deviation on the basis of the same aged seedlings.

First, the amount of light received on the forest floor is directly related to the canopy openness [22], [23]. Due to wind damage (data not shown) and self-thinning (i.e., stem density decreased from 3600 trees ha^−1^ in 1997 to 3383 trees ha^−1^ in 2008), the canopy openness of the unthinned stand increased by 18.37% in 2003 and was close to the canopy openness of the 20% and 30% thinned stands (no significant differences among them). This finding suggested a self-thinning effect in the unthinned stand. Similar increasing trends of canopy openness were found in the 20% and 30% thinned stands, thereby showing that the self-thinning effects also occurred in the less thinned stands. Therefore, the light conditions in the unthinned, 20% and 30% thinned stands were similar to each other from 2003 (6 years after thinning) onward. However, seedlings failed to establish (i.e., no seedling exceeded 1 year old) in the unthinned stand as observed before even the canopy openness was as high as those in the 20% and 30% thinned stands. This finding suggests that besides the light condition (canopy openness), other factors may have more impact on seedling establishment in the unthinned stand, e.g., the deeper litter layer without decomposition and no moss cover (cannot conserve the soil moisture) [3], [5], [32]. Surprisingly, the regenerated seedling density in the unthinned stand was the highest among the four treatment stands in 2003. This can be explained by the presence of more 1-year-old seedlings or current year emerging seedlings in that survey period. At the 11^th^ year after thinning, light conditions in the 20% and 30% thinned stands had improved, but the maximum age of the regenerated pine seedlings was only 3 years, which suggests that it is necessary to further improve the light conditions to enable seedlings to develop into saplings. The light condition in the 50% thinned stand was higher than in the other treatment stands. Consequently, the habitat in the 50% thinned stand is brighter, which is more favourable for pine seedling establishment and growth. The trends in seedling density generally reflect pine regeneration in the 50% thinned stand as reported for the 4^th^ year after thinning treatment at the same site [13]. The canopy openness remains the principal factor in determining the seedling density at the 11^th^ year after thinning, but the pine seedlings needed less light than was previously expected [13]. The growth of pine seedlings in the 50% thinned stand increased with age (**Fig. 5**), but the mean height slowed at the 9^th^ year after thinning. This may suggest that the seedlings in the 50% thinned stand with canopy openness of 25.21% need more light for further development.

Second, soil moisture, which changes largely with the variation in canopy openness [12], [13], is also known to affect the regeneration success of *P. thunbergii* [3], [33]. We only measured the instantaneous soil moistures in August 2003 and October 2008 to confirm the changing trends of soil moisture in the thinning treatments. The results were consistent with the observations at the same site during May 2000 and November 2001 [12]. The higher soil moisture in the 50% thinned stand is important for seedling survival and growth because most of the seedling roots distribute there [7]. The deep moss cover can maintain the soil water [32] and can contribute to the relative soil moisture in the 50% thinned stand, which is likely to provide a more favourable condition for the survival and growth of seedlings. However, the soil moisture in the unthinned stand was the lowest, which suggests that drier condition in the unthinned stand may inhibit the development of seedling roots and may further influence seedling survival. Therefore, only 1-year-old seedlings were observed in the unthinned stand even when the canopy openness was large enough in 2008. Generally, evapotranspiration can influence the characteristics of soil moisture [14]. The evapotranspiration was not directly measured in this experiment, but the lower stem density after thinning should lead to less evapotranspiration in the thinned stands. The forest floor was covered by litter, in particular several years after thinning and by moss in the thinned stands; therefore, the evaporation in the thinned stands may not be affected by the thinning intensity, which can improve the soil moisture as well.

Third, the ground covers (e.g., litter and grass) have negative effects on natural regeneration at the beginning of seedling emergence and also on survival or early growth [7], [34]. The litter in the 50% thinned stand was much less than in the other treatment stands in 2003, but it almost had no change 5 years later in 2008 because of the moss covering the litter (See **Fig. S1**). The litter and moss cover in the 50% thinned stand might impede seedling emergence; therefore, no 1 year or current year seedlings were found during the survey in 2008. The litter cover had similar effects on regeneration as those reported at the 4^th^ year after the thinning treatment at the same site [12]. More moss cover in the 50% thinned stand may be beneficial for conservation of soil moisture and may further favour the survival and growth of seedlings [35]. The grass cover was greater in the 50% thinned stand than in the other treatment stands in both the 2003 and 2008 surveys, which did not seem to affect the survival and growth of the seedlings. This may be because the grass coverage was relatively low (i.e., maximum values were 36.8±25.5% and 35.7±32.3% in 2003 and 2008, respectively, in the 50% thinned stand), and the distribution was uneven.

### Broadleaved Species Regeneration

The natural regeneration of broadleaved tree species indicated that some broadleaved tree species could be established in both thinned and unthinned stands. Furthermore, the trends of regenerated seedling density, age composition and growth for broadleaved species were similar to those of the pine regeneration. However, the regeneration of the tree species was different from each other, i.e., the strong light-demanding species (e.g., *R. pseudoacacia*) dominated regeneration in the 50% thinned stand; the more shade-tolerant species (e.g., *Prunus serrulata*) regenerated in the other treatment stands. Obviously, the successful establishment means that seed sources, seed germination, and seedling emergence and survival should not be obstacles for regeneration of the broadleaved tree species. Of the four regenerated seedling species, *R. pseudoacacia* and *Prunus serrulata* are the planted tree species near the coastal pine forest. The difference in regenerated seedling density was significant (*p*<0.05) between the 50% thinned stand and the other treatment stands (**Table 3**), which suggests that the regeneration of broadleaved tree species requires a similar habitat to pine regeneration. The regenerated seedling density in the 50% thinned stand was approximately twice that in the other treatment stands, which supports the conclusion that canopy openness plays an important role in the survival and establishment of the broadleaved tree species [23], [27]. The regeneration of the broadleaved tree species, especially *R. pseudoacacia,* which seemed to establish successfully in the 50% thinned stand, may have negative consequences for the further development of the regenerated *P. thunbergii* seedlings in the coastal pine forest. For example, in a similar coastal sandy soil, Takeshi *et al*. [14] concluded that *R. pseudoacacia* reduced the light intensity during the growing season and increased the nitrogen content of soil, which resulted in the inhibition of the natural regeneration of *P. thunbergii* [14], [29].

### Implications and Conclusions

In the present study, thinning is a preferred technique to obtain the balance between regeneration and sustainable function, i.e., natural regeneration is successful without inducing wind damage after thinning, at least for the current thinning intensity (50%). Our results verify that thinning with lower intensity (20% and 30% thinned) generally results in the establishment of pine seedlings with a maximum age of 3 years. However, these seedlings could not further develop even though the canopy openness increased through self-thinning. Thinning with higher intensity (50% thinned, which created a canopy openness of more than 30%) can lead to 10-year-old seedlings at the 11^th^ year after thinning. However, the growth rate of the regenerated pine seedlings decreased at the 9^th^ year after thinning. This means that thinning or tending is required for the development of regenerated pine seedlings and for successful pine regeneration in the coastal pine forest. The pine seedling survival and establishment in the unthinned stand cannot be explained by the light condition, which suggests that other environmental factors, such as litter depth, moss cover and soil moisture, or other inhibitions, such as nutrition in the soil, the activity of ectomycorrhizal roots (*P. thunbergii* is an ectomycorrhizal fungal species) [29] and the stand structure characteristics [5], [12], should be considered.

The establishment of some broadleaved tree species was accomplished in the 50% thinned stand and showed the same trend as the pine regeneration. This suggests that if the broadleaved tree species could co-exist with *P. thunbergii*, it would likely form a broadleaved-pine mixed coastal forest. However, the dominant broadleaved tree species that regenerated (*R. pseudoacacia*) is a strong light-demanding tree species. Obviously, it is difficult to harmonise the two light-demanding species in the current coastal pine forest. In addition, *R. pseudoacacia* inhibited the natural regeneration of *P. thunbergii* [13]. Therefore, the development of a broadleaved-pine mixed coastal forest depends on further observations of the development of the broadleaved tree species seedlings.

In conclusion, thinning as a viable silvicultural practice is confirmed for the coastal pine forest from the view of regeneration. The 20% and 30% thinning intensities should be avoided because canopy openness was less than 30%, which severely limited seedling establishment and growth. The thinning that produced more than 30% canopy openness (50% thinned treatment, approximately 1500 stems ha^−1^ at ages of 40–50 years) is suggested to increase natural regeneration in the coastal pine forest because both the seedling density and growth exhibited increasing trends, which, together with the significant moss cover, is vital for conserving the soil moisture in the 50% thinned stand. Further thinning or tending is needed for the development of regenerated pine seedlings when the canopy openness is less than 30%. Both the density and growth of the broadleaved tree species seedlings showed similar trends to those of pine seedlings. However, the development of the broadleaved tree species seedlings needs to be studied further.

## Supporting Information

Figure S1 Regenerated seedling in 50% thinned stand with moss cover at the 11^th^ year after thinning.
(TIF)
