# Supplementary figures and images for: Regeneration of a Coastal Pine (Pinus thunbergii Parl.) Forest 11 Years after Thinning, Niigata, Japan
Source: PLoS One. 2012 Oct 16;7(10):e47593. doi: 10.1371/journal.pone.0047593 (PMC3473025; doi:10.1371/journal.pone.0047593)

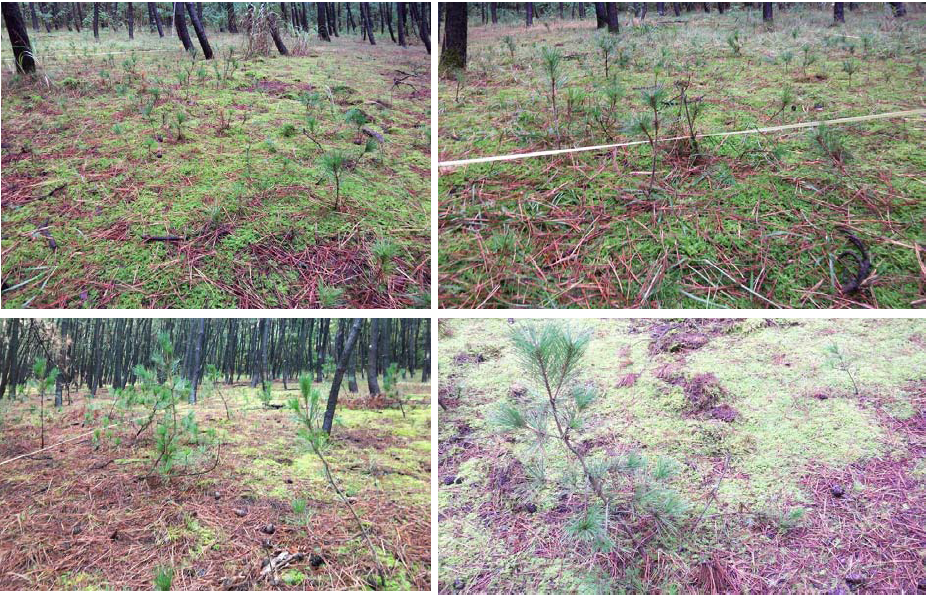

Supplement: Figure S1 — Regenerated seedling in 50% thinned stand with moss cover at the 11th year after thinning. (TIF) [file pone.0047593.s001.tif]
